# A Novel Molecular and Functional Stemness Signature Assessing Human Cord Blood-Derived Endothelial Progenitor Cell Immaturity

**DOI:** 10.1371/journal.pone.0152993

**Published:** 2016-04-04

**Authors:** Oriane Guillevic, Ségolène Ferratge, Juliette Pascaud, Catherine Driancourt, Julie Boyer-Di-Ponio, Georges Uzan

**Affiliations:** INSERM U972, hôpital Paul Brousse, Villejuif, France; Centro Cardiologico Monzino, ITALY

## Abstract

Endothelial Colony Forming Cells (ECFCs), a distinct population of Endothelial Progenitor Cells (EPCs) progeny, display phenotypic and functional characteristics of endothelial cells while retaining features of stem/progenitor cells. Cord blood-derived ECFCs (CB-ECFCs) have a high clonogenic and proliferative potentials and they can acquire different endothelial phenotypes, this requiring some plasticity. These properties provide angiogenic and vascular repair capabilities to CB-ECFCs for ischemic cell therapies. However, the degree of immaturity retained by EPCs is still confused and poorly defined. Consequently, to better characterize CB-ECFC stemness, we quantified their clonogenic potential and demonstrated that they were reprogrammed into induced pluripotent stem cells (iPSCs) more efficiently and rapidly than adult endothelial cells. Moreover, we analyzed the transcriptional profile of a broad gene panel known to be related to stem cells. We showed that, unlike mature endothelial cells, CB-ECFCs expressed genes involved in the maintenance of embryonic stem cell properties such as *DNMT3B*, *GDF3* or *SOX2*. Thus, these results provide further evidence and tools to appreciate EPC-derived cell stemness. Moreover this novel stem cell transcriptional signature of ECFCs could help better characterizing and ranging EPCs according to their immaturity profile.

## Introduction

Endothelial Progenitor Cells (EPCs) have been first isolated from adult peripheral blood in 1997 by Asahara’s team [1]. These cells are mobilized from the bone marrow and are able to integrate vascular structures at neovascularization sites where they differentiate into endothelial cells and proliferate [2]. Many studies have suggested that EPCs are physiologically required for maintaining the vascular integrity [3–5]. *In vitro*, two distinct cell populations derived from EPCs have been identified according to the delay in first colony appearance: early EPCs or Colony Forming Unit-Endothelial Cells (CFU-ECs) and late EPCs or Endothelial Colony Forming Cells (ECFCs), also known as Late Outgrowth Endothelial Cells (Late—OECs) [6]. Both cell populations express specific endothelial markers and have the capacity to stimulate angiogenesis. However, CFU-ECs are actually hematopoietic-derived monocyte/macrophage cell colonies and fail to form vessels *in vivo*. Their pro-angiogenic effect is essentially paracrine. Conversely, ECFCs express specific endothelial markers (CD31, KDR, CD146, CD144 or wWF) but not the hematopoietic and macrophage markers, CD45 and CD14. They display high clonogenic and proliferative potentials compared to CFU-ECs [6–8] and mature endothelial cells [9]. Moreover, ECFCs form stable vessels *in vivo* in different mouse models by incorporating into pre-existing vascular networks [6,10,11]. For these reasons, ECFCs are considered true EPCs progeny with all the phenotypic and functional characteristics of endothelial cells (expression of endothelial- specific markers and vascular reconstruction properties *in vivo*) associated to features of stem/progenitor cells (high clonogenicity and high proliferation rate).

EPCs can be isolated from umbilical cord blood which is a valuable source of stem/progenitor cells. Cord Blood-derived ECFCs (CB-ECFCs) give rise to a greater number of colonies and can be extensively expanded *in vitro* compared to adult peripheral blood-derived ECFCs [12]. In addition, unlike adult vascular endothelial cells, CB-ECFCs have not yet acquired specialized functions. Indeed, we have recently demonstrated that when exposed to appropriate external instructive stimuli, human CB-ECFCs are able to acquire properties of distinct specialized endothelial cells *in vitro*, such as brain microvascular or arterial endothelial cells [13]. These findings suggest that CB-ECFCs retain stem cell features.

While EPCs are a promising cell source for cell therapy, they are still poorly defined and their degree of stemness remains debated. Indeed, EPC immaturity was primarily defined by the expression of the stem/progenitor cell marker CD133 [14,15] but this definition has been recently challenged [16,17]. Over the last years, several studies have shown that cord blood or even adult blood cell subpopulations, which may correspond to (or include) EPCs, express other stem cell markers in addition to CD133. Among these markers, OCT3/4, SOX2, and NANOG represent key components of the core regulatory network governing human embryonic stem cell pluripotency and self-renewal [18–20]. Two studies have shown that CD133^+^ cells from cord blood are also positive for OCT3/4 [21,22]. Moreover Giorgetti *et al*. have shown that early EPCs express, although at much lower levels than hESCs, *OCT3/4*, *SOX2*, *NANOG* and *TDGF1* a subset of pluripotency-associated genes [23]. In 2013, another study has confirmed that early EPCs express NANOG and SOX2, but not OCT3/4 [24]. Furthermore, Lazzari’s team has shown that mature mononuclear cells from adult peripheral blood can also express OCT3/4 [25]. The expression profile of stem cell markers in EPCs remains thus unclear and contradictory.

In this context, and in order to refine the notion of EPC stemness, this study focused on the well-characterized and homogeneous CB-ECFC population. We first quantified the formation of secondary colonies and assessed the generation of induced pluripotent stem cells (iPSCs) as a method to characterize immature CB-ECFCs. Indeed, since their discovery, iPSCs have been generated using numerous somatic cells [26–28]. Interestingly, reprogramming efficiency and kinetics depend on the cell type and immaturity stage [27]. This indicates that somatic cell reprogramming capacity is related to their degree of immaturity. We showed that the efficacy of CB-ECFCs to generate iPSCs is much higher and earlier than that of adult mature endothelial cells (Human aortic endothelial cells, HAECs) and fibroblasts. These ECFC-derived iPSCs were able to differentiate into the three germ layers and to generate functional endothelial cells with an efficiency and kinetics comparable to those of hESCs. Then, to further asses CB-ECFC stemness, we screened a panel of stem cell markers and a transcriptional signature shared by hESCs and CB-ECFCs, but almost undetectable in HAECs, was identified.

Thus, in this study, we demonstrated that CB-ECFCs retain stem cell properties such as a better reprograming potential. Besides, the important number of ECFC-derived iPSCs colonies obtained can represent an effective source of pluripotent stem cells available for pharmaceutical studies. Finally, the expression of this new stemness genes signature could be another criterion to better identify, characterize and range EPCs.

## Materials and Methods

### ECFC Isolation and Culture

Human umbilical cord blood samples were collected in citrate phosphate dextrose solution from healthy full-term newborns. Human samples were collected and handled in compliance with the declaration of Helsinki. Cord blood used for endothelial cell preparation was obtained through a partnership with the Cord Blood Bank of St Louis Hospital (Paris, France) which is authorized by the French Regulatory Authority (authorization N° PPC51) and participates in scientific research. This activity was declared to and authorized by the French Ministry of Research under number AC-2008-376, and to the French Organization for standardization under number 201/51848.1.

ECFCs were isolated as previously described [29]. ECFC colonies appeared after 8 to 12 days of culture. From passage 1 (P1), ECFCs were seeded at 10,000 cells/cm^2^ and grew in EGM-2 MV medium (Lonza, Köln, Germany).

### HAEC Culture

Two samples of primary HAECs from 34 and 23-year old female donors were provided by ATCC (LGC standards, Molsheim, France) and two additional samples from 26 and 28-year old male donors were provided by PromoCell (Heidelberg, Germany). Cells were cultured in EGM-2 MV medium.

### Culture of Undifferentiated Human iPSCs and hESCs

hESCs (H9, H1; WiCell Research Institute, Madison, WI) were cultured according to the supplier’s instructions. Cells were maintained on an irradiated mouse embryonic fibroblast feeder layer in "hESC medium" consisting of Dulbecco’s modified Eagle’s medium/F12 supplemented with 20% Knock Out Serum Replacer (Fisher Scientific, Illkirch, France), 1 mM L-glutamine (Fisher Scientific), 1% penicillin/streptomycin (Fisher Scientific), 0.1 mM β-mercaptoethanol (Fisher Scientific), 0.1 mM nonessential amino acids (Fisher Scientific) and 10 ng/ml recombinant human basic fibroblast growth factor (FGF2, Miltenyi Biotec, Paris, France). The medium was changed daily.

### Senescence-Associated β-Galactosidase Activity Assay

Cells are seeded in P12 well plates at 10,000 cells/cm^2^. At 80% of confluence, senescence-associated β-galactosidase activity is revealed with Senescence Cells Histochemical Staining Kit (Sigma Aldrich, USA) according to the manufacturer’s instructions.

### Clonogenic Frequency Assay

The secondary clonogenic frequency of CB-ECFCs was determined using limiting dilution analysis. ECFCs were isolated from 11 umbilical cord blood samples. Cells from primary colonies were seeded in 96-well plates coated with type I rat tail collagen (Becton Dickinson Biosciences, Sparks, MD), by serial dilutions to obtain 30, 10, 5, 2 and 1 cells per well, with 12 to 36 replicate wells per cell dilution. EGM2-MV medium was changed every 3 days. After 15 days of culture, the number of wells negative for the presence of colony was scored for each dilution, using a phase contrast microscope. The clonogenic frequency was estimated by plotting the median logarithm of the percentage of negative wells according to the number of cells per well. According to Poisson statistics, the 37% logs (1.6) intercept matched with the clonogenic frequency.

### Reprogramming of ECFCs, HAECs and Fibroblasts

Cells were seeded at 10,000 cells/cm^2^ and cultured for 24h in EGM-2 MV medium for ECFCs and HAECs, and in DMEM/F12 supplemented with 10% SVF (S1810, Bio West, FRANCE) for fibroblasts. Cells were transduced with lentiviral vectors RL-EF1-OCT3/4, RL-EF1-SOX2, RL-EF1-KLF4, RL-EF1-v-MYC (Vectalys, Toulouse, FRANCE) with MOI 10 in EGM-2 containing 8 μg/ml polybrene (Sigma-Aldrich, St Louis, MO). Cells were cultured for 7 days in EGM-2 MV medium then transferred and seeded in EGM-2 MV medium for 24h at 40,000 cells/cm^2^ into FIV wells (Fisher Scientific, Illkirch, France) containing irradiated Mouse Embryonic Fibroblasts. The following day, the medium was replaced by hESC medium. IPSC-derived cells appeared between day 11 and 20 and were transferred mechanically every week. Reprogramming efficiency was assessed with the Alkaline Phosphatase Staining Kit II (Stemgent, Cambridge, MA) in one FIV well 12–25 days after transduction depending on the cell type. Efficiency was calculated with 6 samples of ECFCs and 4 samples of HAECs. The number of positive Alkaline Phosphatase (ALP) colonies (red staining) was counted. The efficiency corresponded to the number of ALP positive colonies according to the total number of cells seeded.

### Karyotype Analysis

Chromosomal studies were performed by Tachdjian’s team (Hôpital Antoine Beclerc, Clamart, France). 500,000 iPS cells were blocked at mitotic metaphase after 7 days of growth. The cells were incubated with colchicine 20mg/l (EUROBIO) for 2h at 37°C. Then the cells were trypsinised and centifugated at 1000 rpm for 10 min. A hypotonic shock was attempted with KCL solution (0.075M) at 37°C for 15–20 min followed by a fixation with a methanol/acetic acid solution (3–1 V/V). iPS cells were homogenized in this solution before centrifugation at 1000 rpm for 10 min. This step was repeated 3 times. The samples were stored at 4°C before analysis. 10 cells / passage were analyzed by microscopy.

### *In Vitro* Differentiation of Human iPSCs

For embryonic body (EB) formation, iPSC colonies were harvested by collagenase IV (1mg/ml, Gibco-Life Technologies) and cultured in “EB” medium consisting of Iscove’s Modified Dulbecco’s Medium (IMDM, Invitrogen, Carlsbad, CA) supplemented with 15% FBS (Fetal Bovin Serum, Biological Industries), 1mM L-glutamine, 1% penicillin/streptomycin, 0.1 mM β-mercaptoethanol, 0.1 mM nonessential amino acids (All from Invitrogen) and 10 ng/ml recombinant human basic FGF2 (Miltenyi Biotec, Bergisch Gladbach, Germany).

To induce spontaneous differentiation into the 3 germ layers, cells were transferred after 7 days into fibronectin-coated plates (Sigma Aldrich, St. Louis, MO) and cultured for another 7 days in EB medium. EBs were fixed in 4% ParaFormAldehyde (PFA) + 1XPBS (Electron Microscopy Sciences) and stained with anti-βIII tubulin antibody (R&D, Minneapolis, MN, 1:100), anti-nestin antibody (Millipore, 1:100), anti-α-fetoprotein (AFP) antibody (Santa Cruz, 1:100), anti-Hepatocyte Nuclear Factor-3β (HNF-3β) (Santa Cruz 1:100), anti-CD31 antibody (BD Pharmingen, 1:20 clone WM59) and anti-Smooth Muscle Actin (SMA) antibody (Dako, 1:100).

To induce *in vitro* specific endothelial differentiation, medium was supplemented with 100 ng/ml human stem cell factor (AbCys S.A., Paris, France), 100 ng/ml human Flt3 ligand (AbCys), 10 ng/ml human vascular endothelial growth factor (VEGF-165, PromoKine) and 100 ng/μl BMP4 (AbCys) for 24h. Medium was changed every 3 days. At day 9 of differentiation, EBs were dissociated by collagenase IV (Gibco-Life Technologies) treatment for 1h30 at 37°C. After filtration, dissociated EB cells were labeled with control mouse isotypes or with anti-CD144-PE (Beckman Coulter, Villepinte, France, 1:10, clone TEA 1/31) and anti-KDR-APC (1:5, R&D Systems, clone 89106) and sorted using a FACSDiva cell sorter (Becton, Dickinson and Company, Franklin Lakes, NJ) and plated into fibronectin-coated plates (Sigma-Aldrich) at a density of 15,000 cells/cm^2^ in EGM-2-MV medium.

### Teratoma Formation

ECFC-derived iPSC1 cell line and H9 cells were trypsinized 5 min at 37°C then counted. 100,000 cells were re-suspended in 10μL PBS/BSA and Matrigel^™^ (BD Biosciences, 1:500) solution. For teratoma formation, 8 weeks aged female Severe Combined Immuno-deficient mice (NOD-SCID, IGR, Villejuif, FRANCE) were used. Mice were housed under aseptic conditions with 12-hours light cycle and given tap water sterilized by filtration and gamma-irradiated food ad libitum. The experimental protocol was approved by the Villejuif Institutional Animal Care (SEIVIL, Service d’Expérimentation *in Vivo* Inserm Lavoisier). Two mice were used for iPSC1 cells and 2 other for H9 cells controls. Mice were anesthetized with ketamine (100mg/kg)/xylazine (20mg/kg) injected by the intraperitoneal route. The 10μl of cell suspension was injected intramuscularly. Four weeks after cells injection, teratomas were formed. The mice were anesthetized with ketamine (100mg/kg) / xylazine (20mg/kg) before teratomas collection. The sacrifice was done by cervical dislocation. Each teratoma was fixed overnight in 4% PFA followed by embedding in gelatin/sucrose. 7μm sections were performed that were stained with hematoxylin and eosin (Kit RAL 555, Martillac, France) for histological determinations or with antibodies as described below. These experiments were performed in accordance with the rules established for animal experimentation. A monitoring of the behavior and physiology of the animals has been done every two days. Several parameters were evaluated: weight of the animal, coat appearance, behavior (excessive grooming, defensive state, squinting …), injuries / bites. We planned to euthanize the animals by cervical dislocation during the experiment if they had at least one of the following clinical signs:

- Significant weight loss (from a loss of 20% of initial weight)
- Declaration of infection
- Significant degradation of the animal health status resulting in the accumulation of at least two of the following criteria: poorly maintained peeling or excessive grooming (hair cut away), reduced exploratory behavior, self-injury, poor posture (arched back). If only one of the previously described criteria were observed, analgesics (paracetamol administered orally 1mg/ml) would have been administered to animals in order to prevent any risk of unnecessary pain. In our experiments, no animal presented these signs or died prior the experimental endpoint.

### Flow Cytometry

iPSC-derived endothelial cells were detached with trypsin and immunophenotyping was performed using the following monoclonal antibodies: CD31-FITC (1:25, BD Pharmingen, clone WM59), CD144-PE (1:10, Beckman Coulter, A07481), anti-KDR-APC (1:5, R&D Systems, clone 89106). CB-ECFCs phenotype was assessed with the same antibodies with additional negative controls CD 45-FITC (1:50, Beckman Coulter clone J33) and CD14-FITC (1:50, Beckman Coulter, clone RMO52). Antibodies and matched isotype control (Beckman Coulter) were incubated for 30 min at 4°C. Viability was assessed with 7-AAD (Becton Dickinson). Data were acquired and analyzed on a five-parameter flow cytometer (FACScalibur, Becton Dickinson, San Jose, CA) with Weasel software (WEHI, Melbourne, Australia).

### Immunofluorescence Staining

Cells (ECFCs, ECFC-derived iPSCs) were fixed in 4% PFA/1X PBS (Electron Microscopy Sciences, ref 15714–5) for 10min at room temperature (RT) and washed with 1X PBS. For intracellular staining, cells were permeabilized with 0.2% Triton X100/PBS for 10min at RT. Cells were incubated overnight at +4°C with the following antibodies: anti-Phosphatase Alkaline (PA) (1:40, R&D Systems), anti-OCT3/4 (1:40, R&D Systems), anti-NANOG (1:40, R&D Systems), anti-SSEA4 (1:40, R&D Systems), anti-Tra-160/Tra1-81 (1:200, Abcam), anti-SOX2 (1:40, R&D Systems,), anti-CD31 (1:20, BD Pharmingen, clone WM59) or anti-von Willebrand factor (vWF) (1:300, Dako Cytomation A0082), diluted in 3% BSA/PBS and labeled with Alexa Fluor 488 and 546 Donkey Anti-Goat IgG or Alexa Fluor 488 and 546 Goat anti-Mouse IgG secondary antibodies (Invitrogen, Cergy-Pontoise, France). Cells were then stained with 40,6-diamidino-2-phenylindole (DAPI) and examined with a DMR fluorescence microscope (Leica, Rueil Malmaison, France) equipped with a CoolSnap HQ2 camera (Photometrics, Tucson, AZ) controlled by MetaVue Analyzing Software (Molecular Devices LLC, Sunnyvale, CA).

### Endothelial Function Assays

The expression of the adhesion molecules ICAM-1 and VCAM-1 in response to inflammatory stimuli (TNFα), network formation in Matrigel^™^ and acetylated LDL internalization assays were performed as previously described [31–32].

### RNA Extraction and Reverse Transcription

Total RNA was extracted using RNeasy mini or micro kit (Qiagen, Courtaboeuf, France). Reverse transcriptions were performed with High Capacity cDNA RT Kit (Applied Biosystems, Fischer Scientific, Illkirch, France) according to the manufacturer’s instructions.

### Stemness Gene mRNA Array

Expression of stemness genes was screening in ECFCs at P1 from 3 different cord blood samples with TaqMan Array 96-Well plates “Human Stem Cell Pluripotency” (Applied Biosystems, Fischer Scientific, Illkirch, France). The embryonic stem cell lines H9 at P45 and H1 at P54 (one sample of each) were used as calibrators. Two samples of mature differentiated cells were used as negative controls: One sample of HAECs from a 23-year old female donor and one sample of human skin fibroblasts from a 37-year old female donor, kindly provided by the human cell culture platform of the Myology Institute (Université Pierre et Marie Curie, Paris 6). Quantitative RT-PCRs were performed according to the manufacturer’s instructions using 7300 Real-Time PCR system (Applied Biosystems).

### Quantitative RT-PCR

To confirm the results of the “Human Stem Cell Pluripotency” Array, quantitative Taqman RT-PCR were performed in triplicate on six CB-ECFC samples at P1 according to the manufacturer’s instructions using 7000 Real-Time PCR system (Applied Biosystems). The mean of human embryonic stem cell lines H9 (the same sample at P45 and P52) and H1 (P54) was used as a calibrator for stem cell genes. Four HAEC samples were used at P3 as negative controls and calibrators for endothelial genes. Accession numbers of TaqMan assays are presented in supplemental Data (S1 Table). For the characterization of iPSCs, the expression of endogenous, exogenous stem cell genes and endothelial genes was assessed with SYBR assays using the Stratagene MX3005P system (Agilent Technologies Palo Alto,CA, USA). Primer sequences are shown in S2 Table. The relative quantification of the transcriptional level of each gene was calculated using the 2-[/delta][/delta]Ct method. Endogenous GAPDH was used as a housekeeping gene.

### Statistical Analysis

For statistical analysis, Graphpad Prism 5.0 software was used (Graphpad Software Inc., San Diego, CA). All data are presented as the mean ± SEM. For phosphatase alkaline assay and RT-PCR analysis, one-sample t-tests were used to compare CB-ECFCs and HAECs. Welch’s correction was used when variances were not homogeneous. P values less than 0.05 were considered significant.

## Results

### CB-ECFCs Secondary Clonogenic Frequency

**Fig 1:**
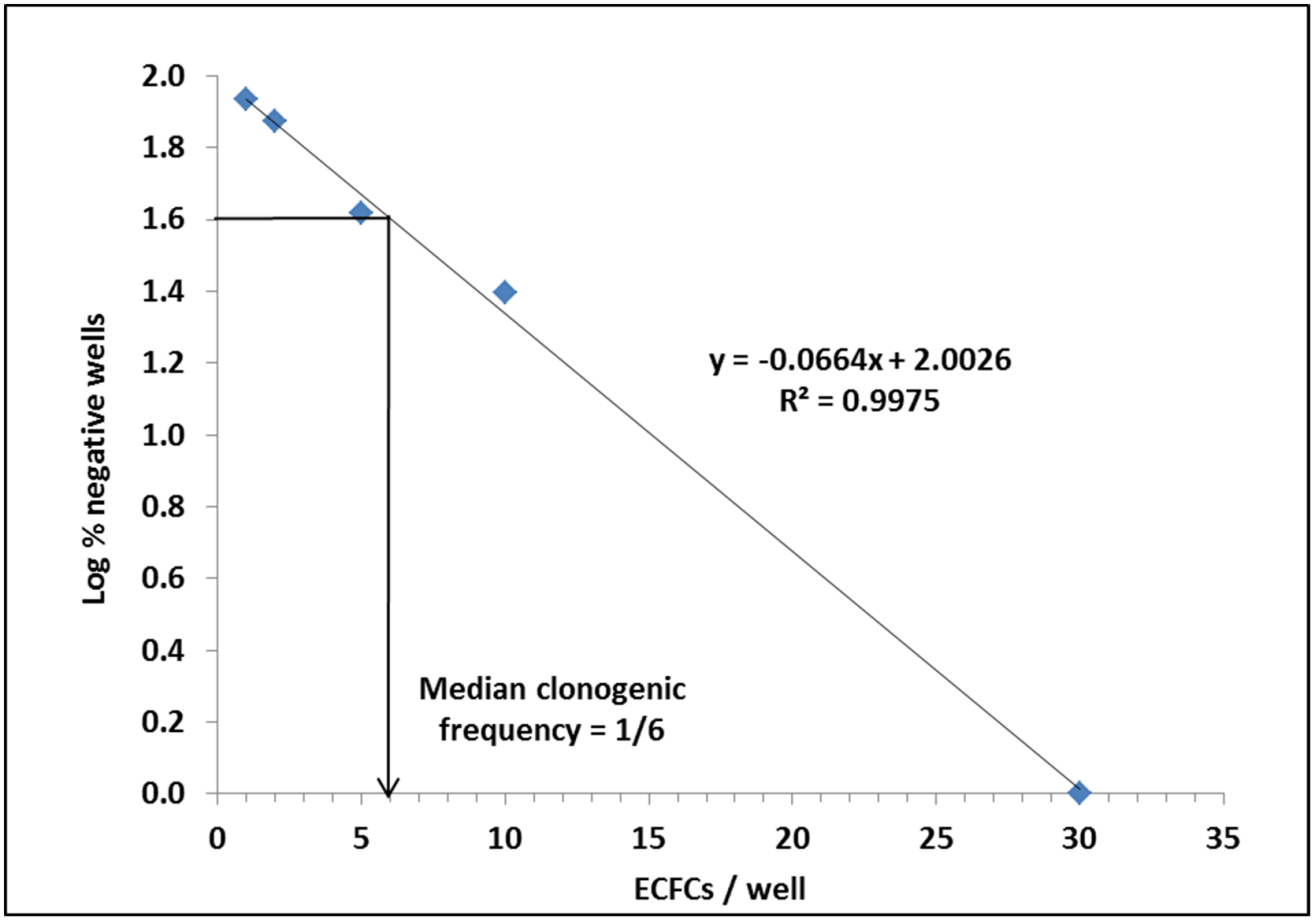
Assessment of human CB-ECFC secondary clonogenicity. Secondary clonogenicity frequency was estimated as 37% log (1.6) intercept.

CB-ECFCs can generate few to more than 100 colonies. For each cord blood sample ECFCs primary colonies were pooled. One part was used for cell amplification and phenotype characterization (S5 Fig), the other part was seeded again to give rise to secondary colonies which clonal growth was assessed by a limiting dilution assay. Results showed that the median secondary clonogenic frequency of CB-ECFCs was equivalent to 1/6, that is to say that, one in 6 cells from the primary colony was able to generate a secondary colony (Fig 1).

### CB-ECFCs Can Be Efficiently Reprogrammed into iPSCs

**Fig 2:**
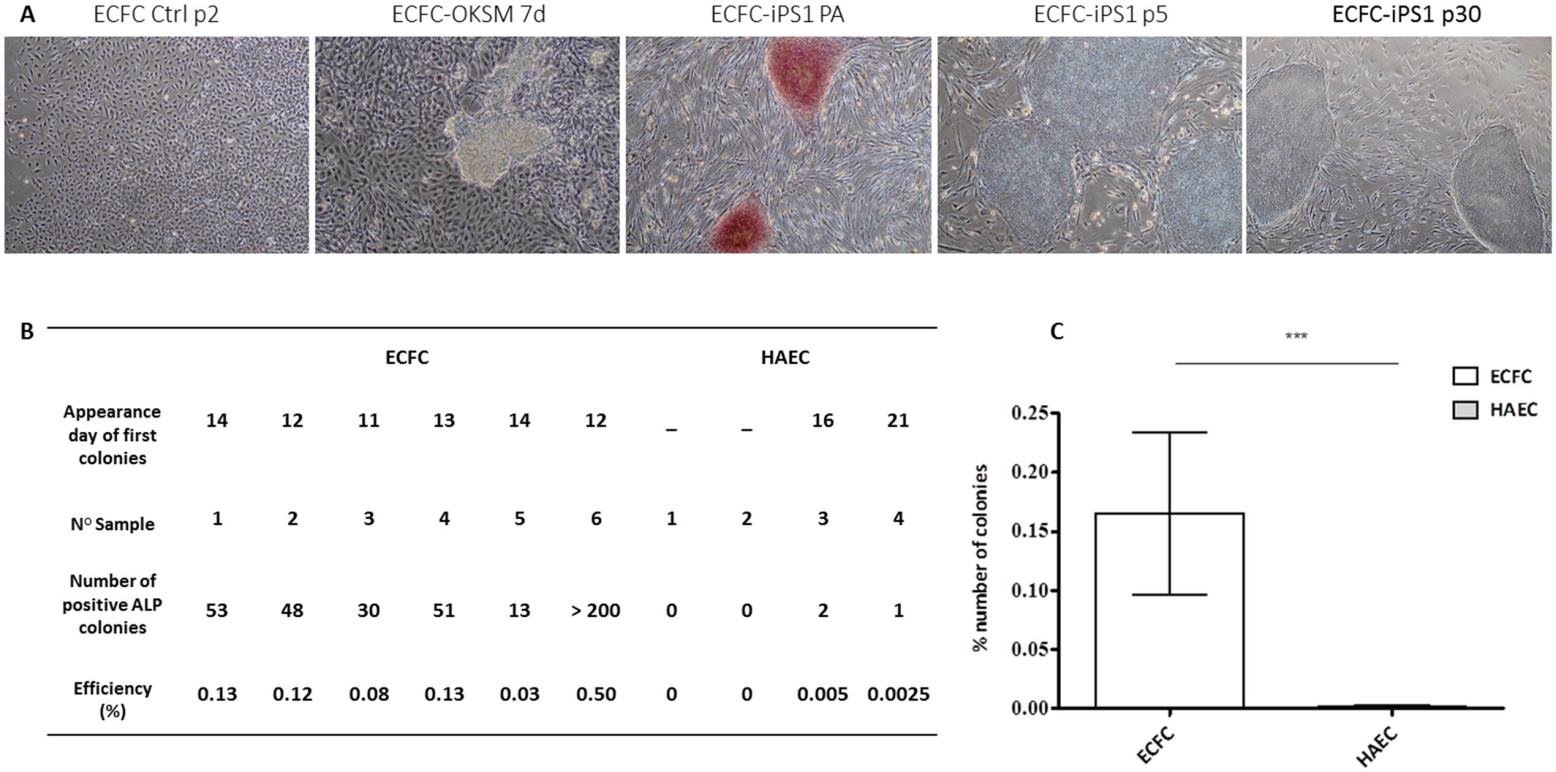
Morphology of ECFC-derived iPSCs and reprogramming efficiency of ECFCs and HAECs. (A) Cobblestone morphology of ECFCs before and 7 days after transduction, ECFC morphology changed and colonies were ALP positives. At passage 5 and 30, ECFC-derived iPSC1 showed typical characteristics of hESC colonies. (B-C) ECFC and HAEC reprogramming efficiency and rate. The reprogramming efficiency was estimated based on the number of ALP positive colonies. Error bars represent SEM (***p < 0.005).

We compare the reprogramming efficacy of CB-ECFCs to mature endothelial cells purified from adult vessels (HAECs). These HAEC samples are derived from young adults. As CB-ECFCs, these cells are functional. Indeed they are able to form several meshes and junctions in Matrigel^™^ assay and they are not senescent (negative in Senescence-associated β-galactosidase activity assay) as showed in supplemental Data (S1 Fig). 40,000 cells were transduced with the lentiviral vectors OCT3/4, SOX2, C-MYC and KLF4 at the same MOI. Transductions were performed on six ECFC samples (2 male and 4 female samples) at P2 and four HAEC samples at P3 or P4. IPSC colonies obtained had a typical ESC-like morphology (Fig 2A). To assess the number of reprogrammed cells, Alkaline Phosphatase (ALP) staining was performed 12–25 days after transduction. ECFC-derived colonies appeared 10–14 days after transduction while HAEC-derived colonies appeared after 15–25 days. The number of colonies with positive ALP red staining was counted (Fig 2A). The mean ECFC reprogramming efficiency was 0.15% corresponding to 48–200 colonies for 40,000 cells seeded. This efficiency was much lower with mean HAECs (0.0018%) (Fig 2B).

### ECFC-Derived iPSCs Show Typical Characteristics of hESCs

**Fig 3:**
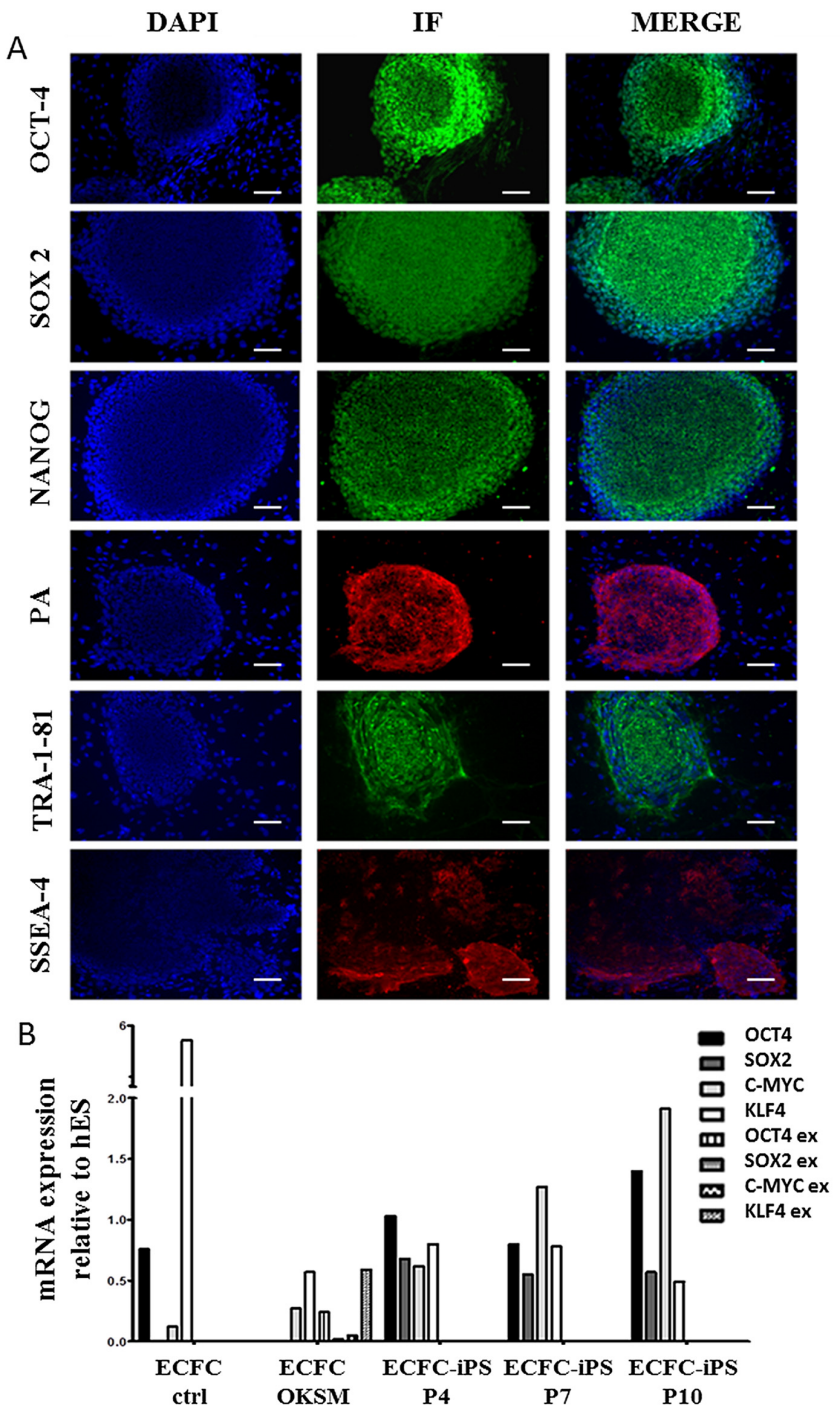
ECFC-derived iPSCs express typical hESC markers. (A) Immunofluorescence analysis of ECFC-derived iPSC1 line. Expression of stem cell markers: OCT3/4, SOX2, NANOG, PA, SSEA4, TRA-1-81. The cells were stained using the 488 and 546-conjugated Donkey Anti-Goat IgG or Goat Anti-Mouse IgG secondary antibody and the nuclei were counterstained with DAPI (blue). Scale bars represent 100 μm. (B) Quantitative RT-PCR analysis of the expression of endogenous (endo) and exogenous (exo) markers: *OCT3/4*, *SOX2*, *KLF4* and *C-MYC* in Ctrl ECFCs, transduced ECFCs (ECFC OKSM) and ECFC-derived iPSC1 at passage 4, 7 and 10. Transcript levels were normalized to GAPDH transcript levels and relative to mean hESCs (H9 samples at P45) as a calibrator.

With the six transductions performed in ECFCs, one iPSC line (ECFC-derived iPSC1) was cultured for up to 30 passages without observing any noticeable differentiation (Fig 2A). This iPSC line was further characterized: karyotype analyses revealed no chromosome abnormalities (46XY) at P10 and P24 (data not shown). Furthermore, this cell line expressed hESC-specific genes and cell surface markers, including NANOG, OCT3/4, SOX2, TRA-1-81 and SSEA4, as shown by immunofluorescence (Fig 3A, negative controls in S2 Fig). Results were confirmed at P4, P7 and P10 by RT-qPCR using specific primers for endogenous or exogenous *OCT3/4*, *SOX2*, *KLF4* and *C-MYC*. Before transduction, ECFCs (Ctrl ECFCs) expressed high levels of *KLF4* and *C-MYC* but very low levels of *SOX2* and *NANOG*. The higher expression of endogenous *KFL4* in ECFC was expected, considering the role of this transcription factor in the induction of key vasoprotective genes in the vascular endothelium [30,31]. Then, at P4, after transduction, a switch was first observed between exogenous and endogenous genes. These latter were similarly expressed in ECFC-derived iPSCs and H9 cell line (Fig 3B). ECFC-derived iPSC1 express other undifferentiated hESC markers such as *NANOG* or *TRA-1-60* (S3 Fig). Before reprogramming, ECFCs expressed the typical endothelial markers C*D144* and *KDR* at high level contrary to the stem cell markers *NANOG* and *TRA-1-60* which are weakly expressed compared to hESCs. After reprogramming, we observe a comparable profile of stemness and endothelial marker between ECFC-iPS cells and hESCs.

**Fig 4:**
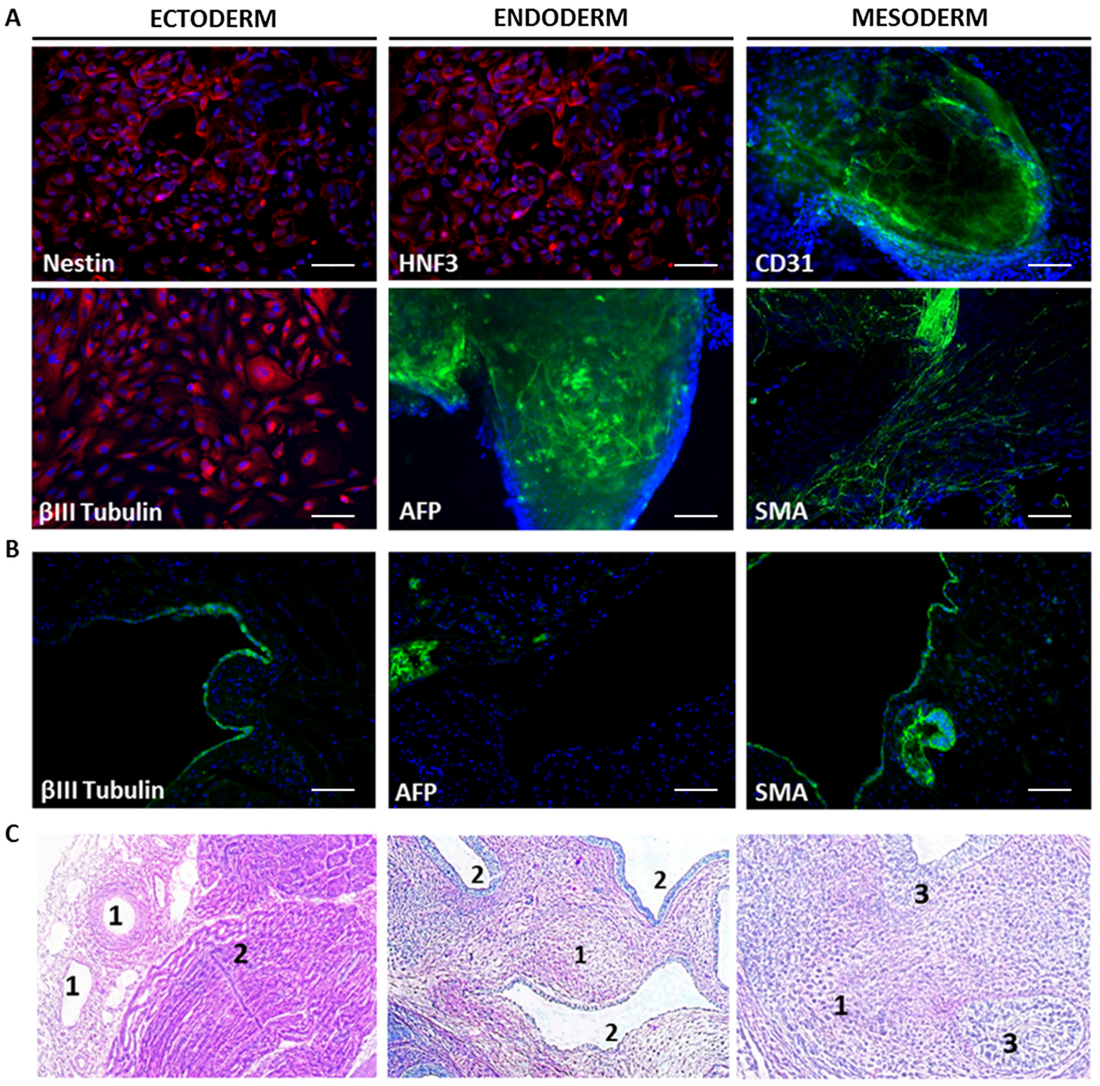
Pluripotency of ECFC-derived iPSCs. (A) Immunostaining of iPSC-derived embryoid bodies revealed the expression of ectodermal (βIII tubulin, nestin), endodermal (AFP, HNF-3β), mesodermal (CD31, SMA) markers. Nuclei were stained with DAPI. Scale bars represent 100μm. (B) Immunostaining of teratomas induced in SCID mice after injection of 100,000 ECFC-derived iPSC1 containing derivatives of the 3 germ layers differentiation (βIII tubulin, AFP and SMA). Nuclei were stained with DAPI. Scale bars represent 100μm. (C) Histology of teratomas (hematoxylin/eosin staining) confirming differentiation into all 3 germ layers: mesoderm (1) endoderm (2) and ectoderm (3).

To form EBs, ECFC-iPS colonies were detached with collagenase and harvested 7 days in low attachment dish. Then EBs were plated in pre-coated gelatin dish with EB medium (iPSC medium without FGF2) for spontaneous differentiation during 7 days (S4A Fig). The expression of markers from the 3 germ lines: endodermal (AFP and HNF-3β), mesodermal (CD31and SMA) and ectodermal (nestin and βIII-tubulin) can be observed in the cells inside the EB (especially for AFP and CD31) or at its edge (Fig 4A and S4B Fig).

Transplantation into immunodeficient SCID mice resulted in the formation of typical teratomas containing derivatives of the 3 germ layers which were revealed by immunostaining. The hematoxylin/eosine staining revealed mesenchyme tissue with stellate cells dispersed in myxoid stroma, endoderm layer with glandular epithelium specific structure. We can also observe specific structure of ectoderm germ layer with multilayered Malpighi epithelium (Fig 4B and 4C).

### iPSC Endothelial Differentiation

**Fig 5:**
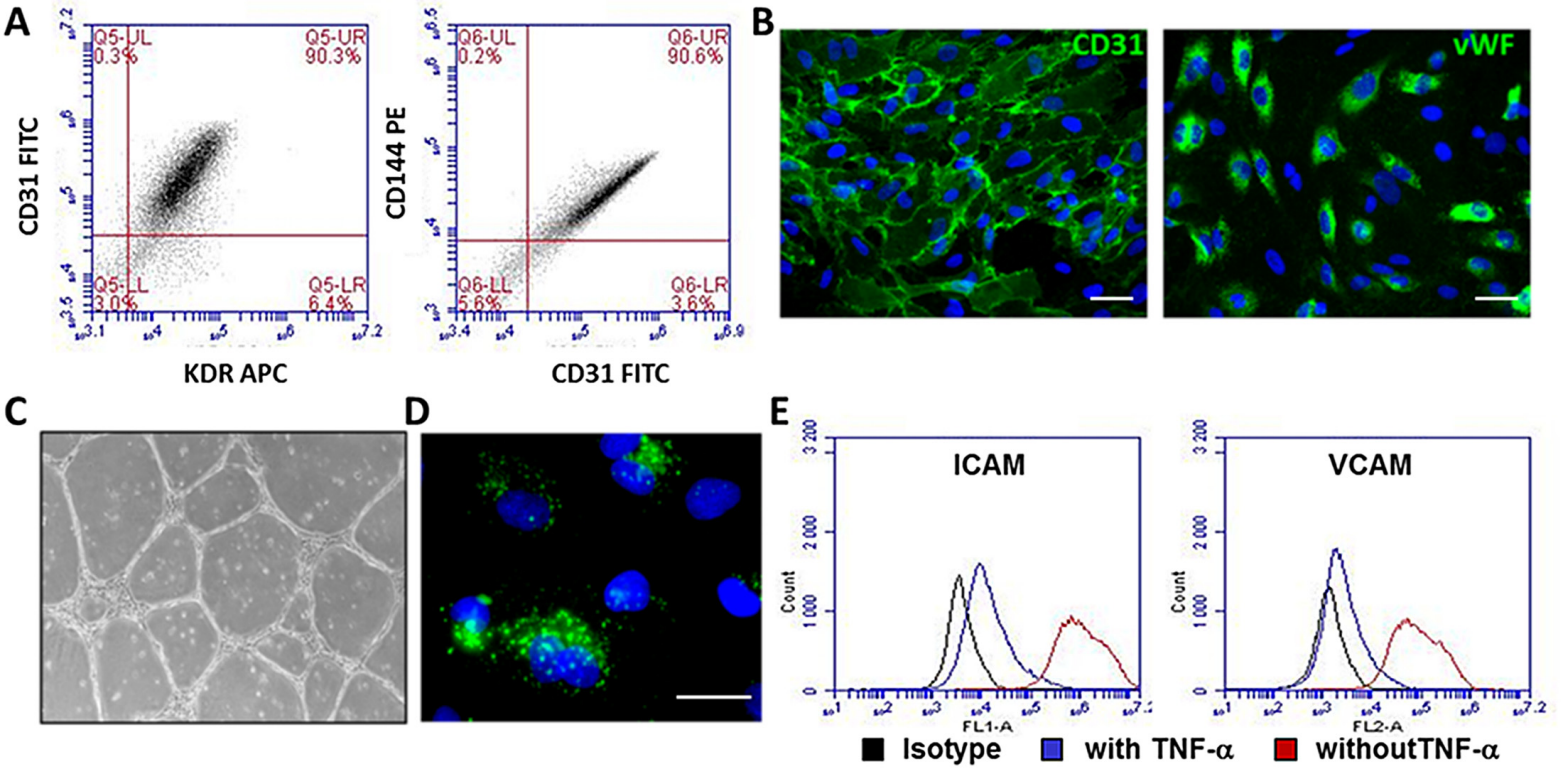
Phenotypic and functional characterization of iPSC-derived endothelial cells. (A) Expression of specific endothelial markers (KDR, CD31 and CD144) by flow cytometry at passage P4. (B) CD31 and vWF immunofluorescent staining at passage 5. Scale bars represent 50μm. (C) Vascular-like network structures after 24h onto Matrigel^™^ at passage P4. (D) Representative diacetylated low-density lipoprotein incorporation at passage P4. Scale bars represent 25μm. (E) TNF-α upregulated *ICAM-1* and *VCAM-1* at passage P5.

Endothelial differentiation was induced by adjusting the protocol developed by Goldman *et al*. for hESCs [32]. As shown in Fig 5A, at P3, more than 90% of cells expressed the specific endothelial cell surface markers CD144, CD31 and KDR. Immunofluorescence microscopy showed characteristic CD31 and vWF expression on most cells. (Fig 5B). They formed typical vascular-like network structures on Matrigel^™^ (Fig 5C). These cells were also able to uptake diacetylated low-density lipoproteins (Fig 5D) and were activated in response to the pro-inflammatory factor TNF-α, as shown by the upregulation of the intercellular adhesion molecules ICAM-1 and VCAM-1 (Fig 5E). These results confirm that this ECFC-derived iPSCs is able to differentiate into functional endothelial cells.

### CB-ECFCs Express Stemness Genes

We hypothesized that the efficient reprogramming capacity of CB-ECFCs was related to their relative stemness. Thus, to assess their immaturity, RT-qPCR pre-screening of 92 genes chosen according to a study of the International Stem Cell Initiative was performed on three CB-ECFC samples. For this screening, two hESC lines (H1 and H9) were used as positive controls and two types of human adult differentiated cells (HAECs and fibroblasts) as negative controls.

As expected, CB-ECFCs and HAECs strongly overexpressed the endothelial markers *PECAM-1/CD31* (platelet endothelial cell adhesion molecule) and *CDH5* (cadherin 5, type 2 (vascular endothelium), *VE-cadherin*, *CD144*) compared to H1 and H9 hESC lines and fibroblasts. *CD34* was also highly expressed in both endothelial cell populations, with a greater (almost 1 Ct difference) but heterogenic transcription level in CB-ECFCs. Among all the genes expressed, only those with a minimum Ct difference of 0.5–1 between CB-ECFCs and mature differentiated cells were used to determine a differential transcriptional stemness signature. Thus, 9 genes were selected: *DNMT3B* (DNA (cytosine-5-) -methyltransferase 3β), *GBX2* (gastrulation brain homeobox 2), *GDF3* (growth differentiation factor 3), *GRB7* (growth factor receptor-bound protein 7), *ISL1* (ISL LIM homeobox 1), *PODXL* (Podocalyxin), *NANOG*, *SOX2* (SRY (Sex determining Region Y)-box 2) and *TDGF1* (teratocarcinoma-derived growth factor 1). Interestingly, *OCT3/4*—*POU5F1* (POU class 5 homeobox 1) expression was equivalent in CB-ECFCs and differentiated cells (Table 1).

**Table 1:** Stemness genes expression array. Data are expressed as ΔCt. Transcript levels were normalized to GAPDH transcript level. Genes whose detection threshold is over 35 cycles are considered as not expressed and symbolized by “X”.

	hES		ECFC				Differentiated cells	
	*H1*	*H9*	*sample 1*	*sample 2*		*sample 3*	*HAEC*	*fibroblasts*
**Endothelial &/or ECFCs markers**								
**CD34**	12.72	14.29	3.06	8.07	4.80		7.14	13.17
**CDH5**	13.75	15.97	1.67	2.23	2.17		2.04	12.81
**PECAM-1**	13.02	17.43	1.39	2.10	1.44		1.59	15.96
**PODXL**	1.85	1.35	2.94	5.22	4.36		5.07	6.31
**Pluripotent markers &/or maintenance of pluripotency**								
**DNMT3B**	1.93	2.36	8.76	9.51	9.05		9.72	10.82
**GDF3**	7.90	7.80	9.89	9.42	10.55		11.06	X
**NANOG**	5.28	4.13	16.39	13.26	16.96		17.40	19.85
**POU5F1**	10.42	8.17	14.01	15.14	13.76		13.04	14.14
**SOX 2**	6.38	5.87	15.15	16.00	20.60		X	X
**TDGF1**	3.95	3.42	12.38	14.91	19.51		18.60	18.67
**Stemness markers**								
**GBX2**	9.56	10.64	11.78	11.16	11.66		12.44	19.20
**GRB7**	7.52	7.22	14.42	15.41	17.59		16.71	16.59
**Early differentiation marker**								
**ISL1**	10.84	12.98	15.01	13.54	X		18.63	20.29

**Fig 6:**
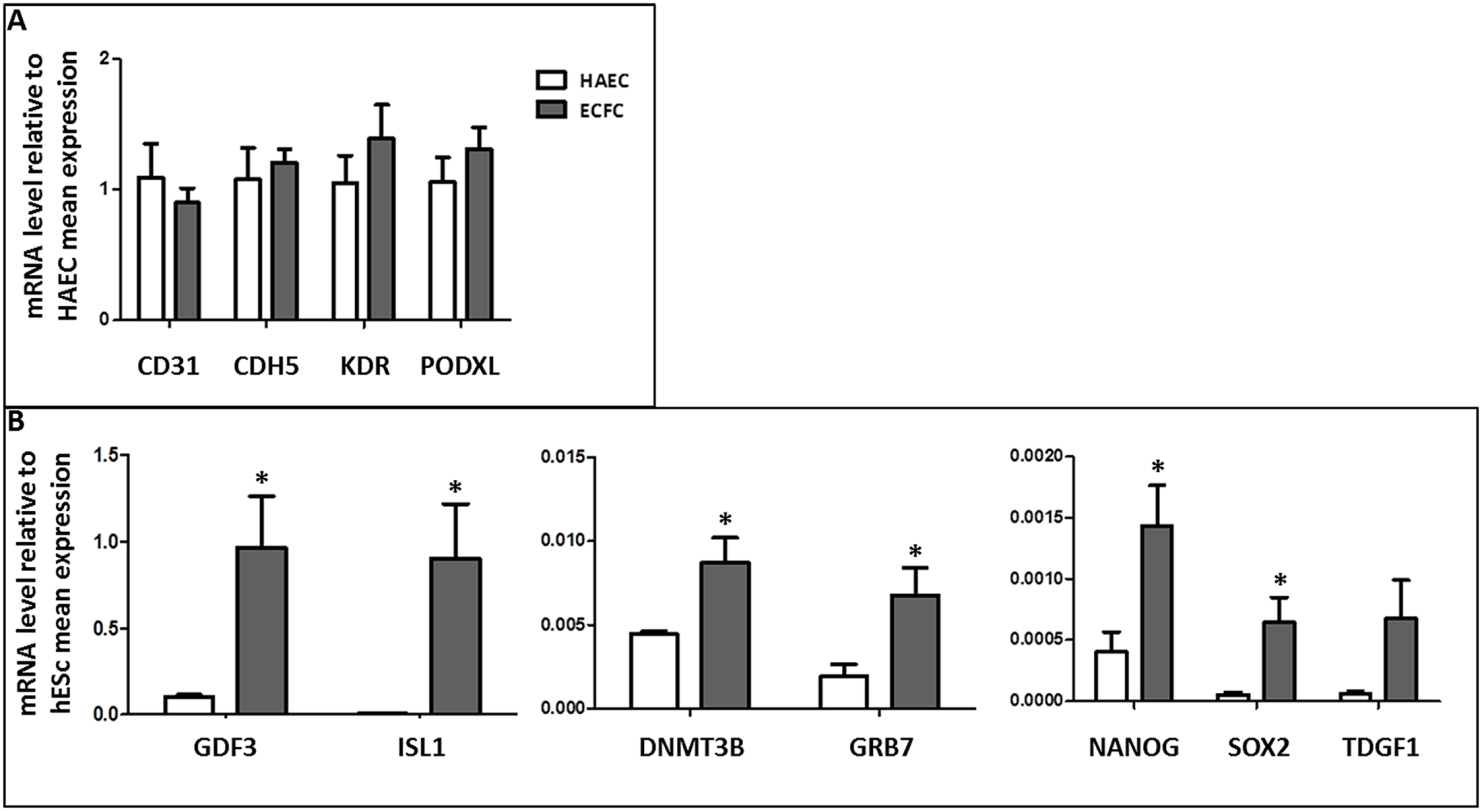
Endothelial and Stemness transcriptional signature of CB-ECFCs. (A) Transcript levels of endothelial markers. Quantitative RT-PCR analysis in ECFCs at passage P1 (n = 6) and adult HAECs at passage P3 (n = 4). Transcript levels were normalized to GAPDH transcript levels and relative to mean HAECs. (B) Transcript levels of stemness markers. Quantitative RT-PCR analysis in ECFCs at passage P1 (n = 6) and adult HAECs at passage P3 (n = 4). Transcript levels were normalized to GAPDH transcript levels and relative to mean hESCs (2 H9 samples at P45 and P52 and 1 H1 sample at P54). Error bars represent SEM (*p < 0.05).

To confirm this stemness genes pattern, RT-qPCRs were performed on six samples of CB-ECFCs and four samples of adult HAECs. CB-ECFCs had a strong endothelial profile (*CD31*, *CDH5* and *KDR/VEGFR-2* (Vascular Endothelial Growth Receptor 2), similar to the mature endothelial cells HAECs (Fig 6A). Note that *PODXL* cannot distinguish CB-ECFCs from HAECs. CD34 mRNA level was also high, but very variable, in the 2 cell populations without significant difference between CB-ECFCs and HAECs (data not shown). This endothelial phenotype was associated with the corroborated expression of the stemness genes *DNMT3B*, *GDF3*, *GRB7*, *NANOG*, and *SOX2*, and of the early mesodermal marker *ISL1* (Fig 6B). The differential expression of *GBX2* (data not shown) and *TDGF1* was not statistically relevant between ECFCs and HAECs. *GDF3* and *DNMT3B* had the highest transcriptional level in CB-ECFCs with a detection threshold at 27–28 Ct. Ct of the other genes ranged between 33 and 35. For all selected genes, expression levels were 2–10 times higher in CB-ECFCs than in HAECs for which detection thresholds were over 35 Ct, except for *GDF3* and *DNMT3B*. Moreover, *ISL1*, *SOX2* and *TDGF1* were almost undetectable in HAECs. Interestingly, *GDF3* and *ISL1* mRNA levels were similar in CB-ECFCs and hESCs whereas *DNMT3B* and *GRB7* levels were 100–200 lower. *NANOG*, *TDGF1* and *SOX 2* levels were 1000–2000 times lower than in H1 and H9 lines.

## Discussion

ECFCs represent a canonical cell type derived from EPCs in culture characterized by clonogenic, proliferative and pro-angiogenic potentials. We have recently shown that these progenitor cells can acquire, under specific conditions, specialized endothelial phenotypes and functions similar to those of arterial or cerebral microvascular endothelial cells [13]. This finding shows the relative plasticity of ECFCs, probably related to their stem cell/progenitor nature. Interestingly, this immaturity seems to play a central role in their functionality. Indeed, cord blood EPC-derived ECFCs are more clonogenic and proliferative than adult cells, they present a high telomerase activity [12] and they are able to form functional long-lasting vessels, unlike those formed by adult ECFCs which rapidly regress [10]. Nevertheless, the concept of EPC stemness remains unclear and poorly documented. EPC immaturity is essentially defined by the expression of the stem/progenitor cell markers CD133 but its expression by CB-ECFCs is reassessed [16,17] and CD34 which also expressed on hematopoietic stem and progenitor cells [33,34]. That is why a key issue of this study was to highlight new stemness markers.

Beyond the stemness issue, the lack of consensus about EPC markers raises questions about the strength of several studies on the angiogenic potential of EPCs, which are only based on extrapolation from non-purified populations, sorted using controversial and non-exclusive markers such as CD133/AC133 or CD34. For this reason, in order to refine the notion of EPC stemness, this study focused on the well-characterized and homogeneous CB-ECFC population, isolated after a limited number of passages. These immature cells were tested for secondary colony formation, iPSC generation and the expression of a dedicated panel of genes involved in stemness.

Clonogenicity is one of the hallmarks of stem cell and progenitor cells. ECFCs can generate primary, secondary and even tertiary colonies. In order to quantify this property, a limiting dilution analysis and a Poisson’s representation, similar to that used to quantify the clonogenicity of hematopoietic stem/progenitor cells, were used. One in 6 cells is able to generate a secondary colony. This results shows that CB-ECFCs contain highly clonogenic subpopulations.

To further assess ECFC immaturity, their efficiency in generating iPSCs was tested. Since the discovery of somatic cell reprogramming into pluripotent cells, iPSCs generation has been induced from numerous somatic cells with variable kinetics and efficiencies. It is assumed that the differentiation state of cells could influence reprogramming efficiency. Indeed, in 2009, Eminli *et al*. have demonstrated that hematopoietic progenitors were more efficiently reprogrammed into iPSCs (7%-28%) than differentiated blood cells (0.02%-0.6%) [27]. We used the efficacy of iPSCs generation as a method to define the degree of ECFC immaturity. The ability of immature cord blood-derived ECFCs and mature endothelial cells (HAECs) to be reprogrammed into iPSCs was compared using the Yamanaka’s standard protocol. The efficiency of CB-ECFCs to be reprogrammed was at least 80 times higher and 1.5–2 times faster than that of HAECs. Two fibroblast samples were used as controls and results similar to those of HAECs were obtained (data not shown). These results further demonstrate the immature nature of ECFCs.

In order to confirm that ECFCs were able to generate genuine iPSCs, 1 cell line, ECFC-derived iPSC1, was analyzed. This cell line was stable for up to 30 passages. It expressed genes involved in stemness (*OCT3/4*, *SOX2* and *NANOG*), was able to generate teratoma in immunodeficient SCID mice and to differentiate into early mesodermal, endodermal and ectodermal cells as shown by the expression of specific markers. In addition to this ability to differentiate spontaneously into three early germ layers, this iPSC line can be fully differentiated into a defined cell type as functional mature endothelial cells.

We then hypothesized that the high reprogramming efficiency displayed by ECFCs could be related to the constitutive expression of genes involved in stem cell biology. The stem cell gene transcriptional pattern of CB-ECFCs, adult HAECs and fibroblasts were thus compared using hESC lines as a positive control. A first screening was performed, including 92 genes chosen according to an international study involving 17 laboratories to determine a homogeneous stemness feature shared by 59 hESC lines [35]. Among the selected genes, some are considered stem cell makers while others are differentiation markers. As expected, endothelial markers (*PECAM-1*, *CDH5* and *CD34*) were highly expressed in ECFCs and HAECs unlike hESCs and fibroblats. Morever, CB-ECFCs expressed the sialoprotein Podocalyxin described as a hemangioblastic and endothelial marker [36–38]. Among the differentiation markers, a low and heterogenic but differential expression of the early mesodermal marker *ISL1* was also observed in cord blood-derived ECFCs compared to adult HAECs and fibroblasts. This first screening revealed the overexpression of 7 stem cell genes in ECFCs compared to adult differentiated cells: *DNMT3B*, *GBX2*, *GDF3*, *GRB7*, *NANOG*, *SOX2* and *TDGF1*. Most of these genes are part of the molecular signature of hESC lines [39]. Surprisingly in ECFCs, HAECs and fibroblasts, a low but comparable transcriptional level of *OCT3/4*, one of the most important marker of embryonic stem cells and involved in self-renewal and pluripotency [40], was detected. Our results are consistent with a study reporting that *OCT3/4* is also expressed in somatic cells as peripheral blood mononuclear cells [25]. This previous pattern of stem cell genes was then confirmed by RT-qPCR using additional cell samples. CB-ECFCs present both a strong endothelial profile associated with the expression of stemness-related genes consisting in the 5 confirmed markers *DNMT3B*, *GDF3*, *GRB7*, *NANOG*, and *SOX2*. Expression of the early mesodermal differentiation marker *ISL1* was also confirmed. The highest transcription levels were observed for *GDF3* and *DNMT3B* in ECFCs. All these genes were 2–10 times more expressed in ECFCs than in HAECs.

Interestingly, *GDF3* and *ISL1* transcription levels in CB-ECFCs reach those measured in hESCs. GDF3, a member of the TGFβ superfamily [41] is expressed in pluripotent cells and is down-regulated during differentiation [42]. It maintains hESCs in an undifferentiated state through inhibition of the BMP pathway [43]. *In vitro*, progenitors which expressed *ISL1*, an early mesodermal differentiation marker, can differentiate into endothelial cells, cardiomyocytes and smooth muscle cells [44] showing their multipotent nature. In 2014, Barzelay *et al*. have first reported that *ISL1* expression in endothelial cells results in their enhanced proliferative, paracrine, migratory and adhesion properties as well as an enhanced vascularization in mice [45]. *ISL1* expression could provide ECFCs with both stemness and endothelial properties. It would be interesting to study the correlation between *ISL1* expression level and their plasticity and clonogenicity capacities. The transcriptional levels of the other genes were very low compared to hESCs. However, *DNMT3B* was one of the most expressed stem cell gene in ECFCs. This DNA methyltransferase does not seem to be involved in self-renewal, but rather in optimal mesodermal or hematopoietic differentiation [46–48]. Moreover, *DNMT3B* affects *OCT3/4* and *NANOG* expression by methylating their promoters during mouse embryonic cell differentiation [49]. The role of *GRB7* in embryonic stem cells remains unclear and few studies have shown its involvement in stem cell functions. This adaptor protein can interact with *c-kit* [50], a receptor involved in various cell functions such as mitogenesis, chemotaxis, survival or differentiation. It also participates in the regulation of cell mobility and angiogenesis [51]. The two remaining genes, *NANOG* and *SOX2*, play central roles in hESCs. They are considered, together with *OCT3/4*, master transcriptional factors. They collaborate in autoregulatory and feedback loops maintaining pluripotency and self-renewal in mouse and human ESCs [20]. Interestingly, *NANOG* is also involved in endothelial cell physiology. Indeed, Kolher *et al*. have demonstrated that human umbilical vein endothelial cells (HUVECs) and mature endothelial cells may express *NANOG* at low level. Moreover, this study has shown that, in HUVECs, the transcription of *FLK*-1 (*VEGFR-2 /KDR)*, a major regulator of endothelial cell function, is regulated by the binding of *NANOG* to its promoter [52].

Thus, all these genes are involved in stem or progenitor cell functions and some of them also play a role in mature endothelial cells. Moreover, several studies have shown that these different genes could interact with each other. Finally, this study defines a transcriptional signature specific to cord blood-derived ECFCs, which takes into account the dual nature of these cells which present both endothelial and stem/progenitor features.

## Conclusion

Assessing EPC immaturity remains a key issue to characterize these progenitors. In this study, we provided further evidence and tools to appreciate ECFC stemness, an *in vitro* cell population derived from cord blood EPCs. We showed that these cells have a high clonogenic potential and can be very efficiently reprogrammed into iPSCs which may represent a useful source of pluripotent stem cells available for pharmaceutical studies. These particular capacities reflect the immature nature of CB-ECFCs. Moreover, we defined a novel stem cell transcriptional signature of CB-ECFCs distinguishing them from adult mature endothelial cells. This involves *DNMT3B*, *GDF3*, *GRB7*, *NANOG* and *SOX2*. However, the specific role of these genes has to be determined to define their precise involvement in stem cell and/or endothelial properties of EPCs, this dual nature being required for their physiological function in vascular repair.

## Supporting Information

S1 Fig Functional and Senescence *in vitro* assays. (A) *In vitro* Matrigel^™^ Assay. Photographs are representative for the tube-like network structures shown after 24 hours onto Matrigel^™^. Scale bars represent 200μm. (B) Senescence-associated β-galactosidase activity assay. Photographs illustrating the presence or not of senescent cells (blue) in early cord blood ECFCs, late cord blood ECFCs and HAEC samples. Scale bars represent 100μm.
(TIF)

S2 Fig Negative Controls of staining. The ECFC-iPS1 colonies were incubated directly with the 488 and 546-conjugated Donkey Anti-Goat igG secondary antibodies (respectively A and C) or with the 488 and 546-Goat Anti-Mouse igG secondary anti bodies (B and D). Scale bars represent 100μm.
(TIF)

S3 Fig Evolution of stem cell and endothelial markers expression before and after CB-ECFCs reprogramming. Quantitative RT-PCR analysis of the stem cell markers *NANOG* and *TRA-1-60*, and the endothelial markers *CD144* and *KDR* expression in Ctrl ECFCs, transduced ECFCs (ECFC OKSM) and ECFC-derived iPSC1 at passage 4, 7 and 10. Transcript levels were normalized to GAPDH transcript levels and relative to mean hESCs (H9 samples at P45) as a calibrator.
(TIF)

S4 Fig EBs morphologies and staining after 7 days of differentiation. (A) EBs formation after 7 days in ultra-low attachment dish and after 7 days on gelatin with the different morphologies of cells. Scale bars represent 100μm. (B) Immunostaining of iPSC-derived embryoid bodies: Expression of ectodermal (βIII tubulin, nestin), endodermal (AFP, HNF-3β) and mesodermal (CD31, SMA) derivatives. Scale bars represent 50μm.
(TIF)

S5 Fig CB-ECFCs phenotype. Representative Flow cytometry analysis of the positive endothelial markers CD31, CD144 and KDR (A) and of the negative hematopoetic/monocytic markers CD45 and CD14 (B) (IgG isotopic control: black line, markers: red line).
(TIF)

S1 Table Accession numbers of TaqMan^®^ (Applied Biosystems) assays used for quantitative-PCR. (DOCX)

S2 Table Primer sequences of endogenous, exogenous and endothelial genes used for SYBR assays. (DOCX)
