# Supplementary figures and images for: A Novel Molecular and Functional Stemness Signature Assessing Human Cord Blood-Derived Endothelial Progenitor Cell Immaturity
Source: PLoS One. 2016 Apr 4;11(4):e0152993. doi: 10.1371/journal.pone.0152993 (PMC4820260; doi:10.1371/journal.pone.0152993)

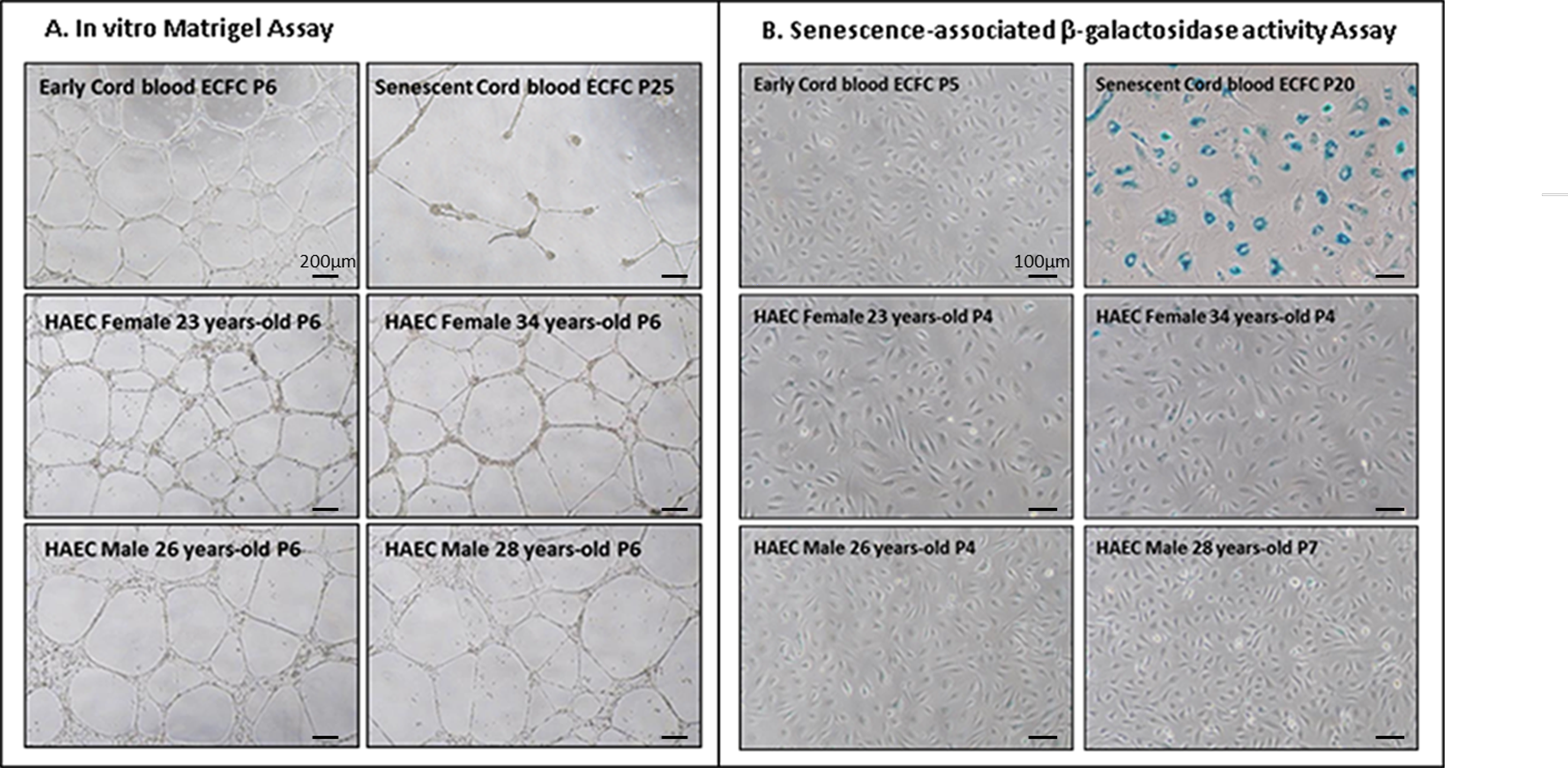

Supplement: S1 Fig — (A) In vitro Matrigel™ Assay. Photographs are representative for the tube-like network structures shown after 24 hours onto Matrigel™. Scale bars represent 200μm. (B) Senescence-associated β-galactosidase activity assay. Photographs illustrating the presence or not of senescent cells (blue) in early cord blood ECFCs, late cord blood ECFCs and HAEC samples. Scale bars represent 100μm. (TIF) [file pone.0152993.s001.tif]

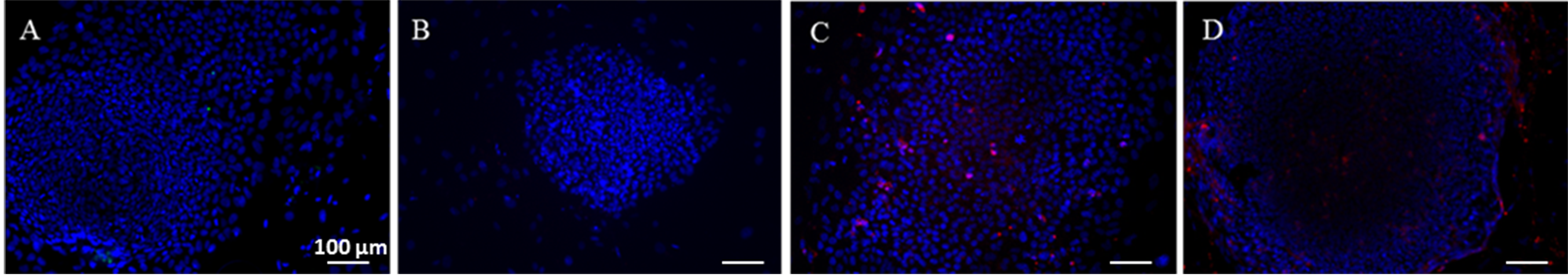

Supplement: S2 Fig — The ECFC-iPS1 colonies were incubated directly with the 488 and 546-conjugated Donkey Anti-Goat igG secondary antibodies (respectively A and C) or with the 488 and 546-Goat Anti-Mouse igG secondary anti bodies (B and D). Scale bars represent 100μm. (TIF) [file pone.0152993.s002.tif]

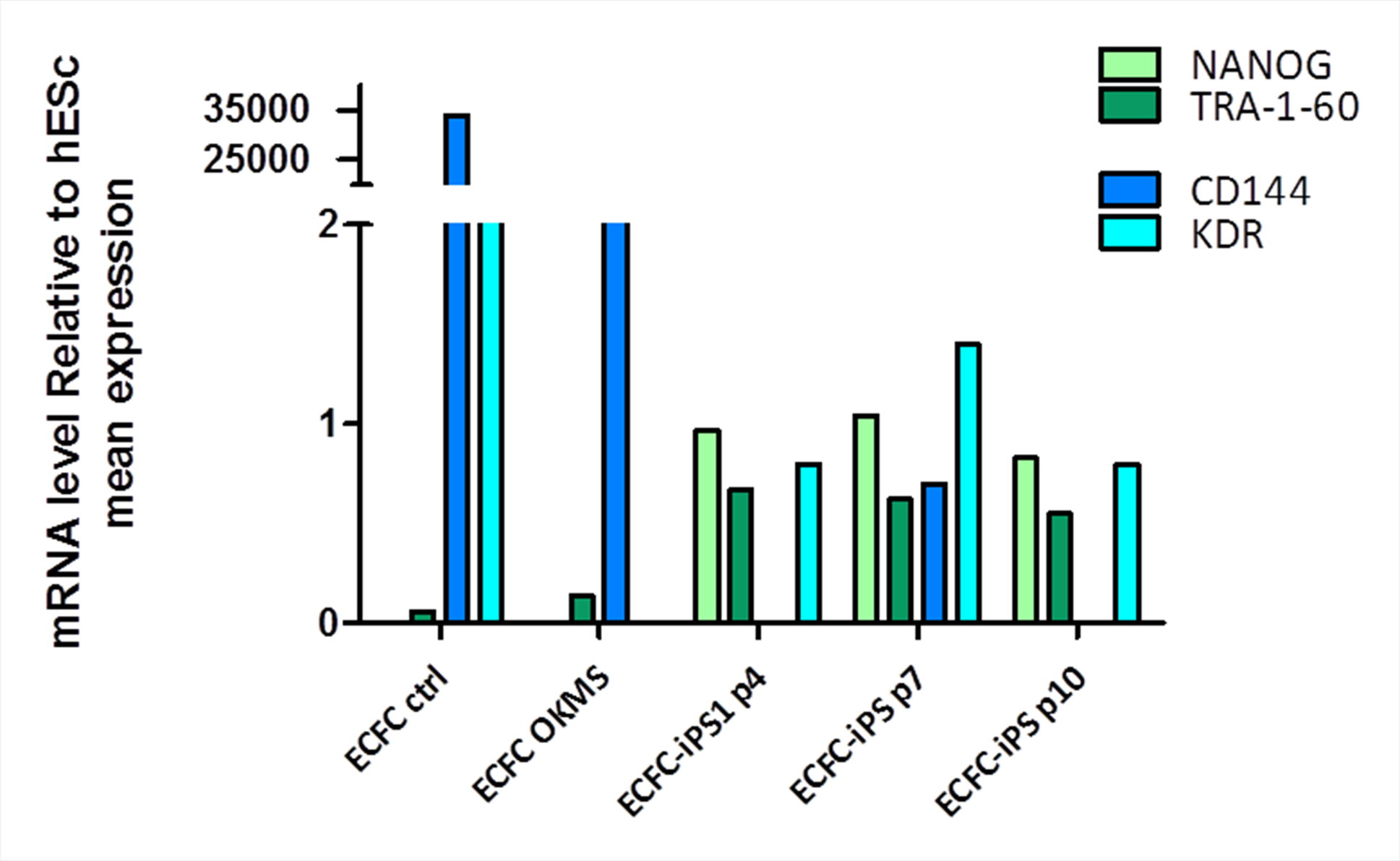

Supplement: S3 Fig — Quantitative RT-PCR analysis of the stem cell markers NANOG and TRA-1-60, and the endothelial markers CD144 and KDR expression in Ctrl ECFCs, transduced ECFCs (ECFC OKSM) and ECFC-derived iPSC1 at passage 4, 7 and 10. Transcript levels were normalized to GAPDH transcript levels and relative to mean hESCs (H9 samples at P45) as a calibrator. (TIF) [file pone.0152993.s003.tif]

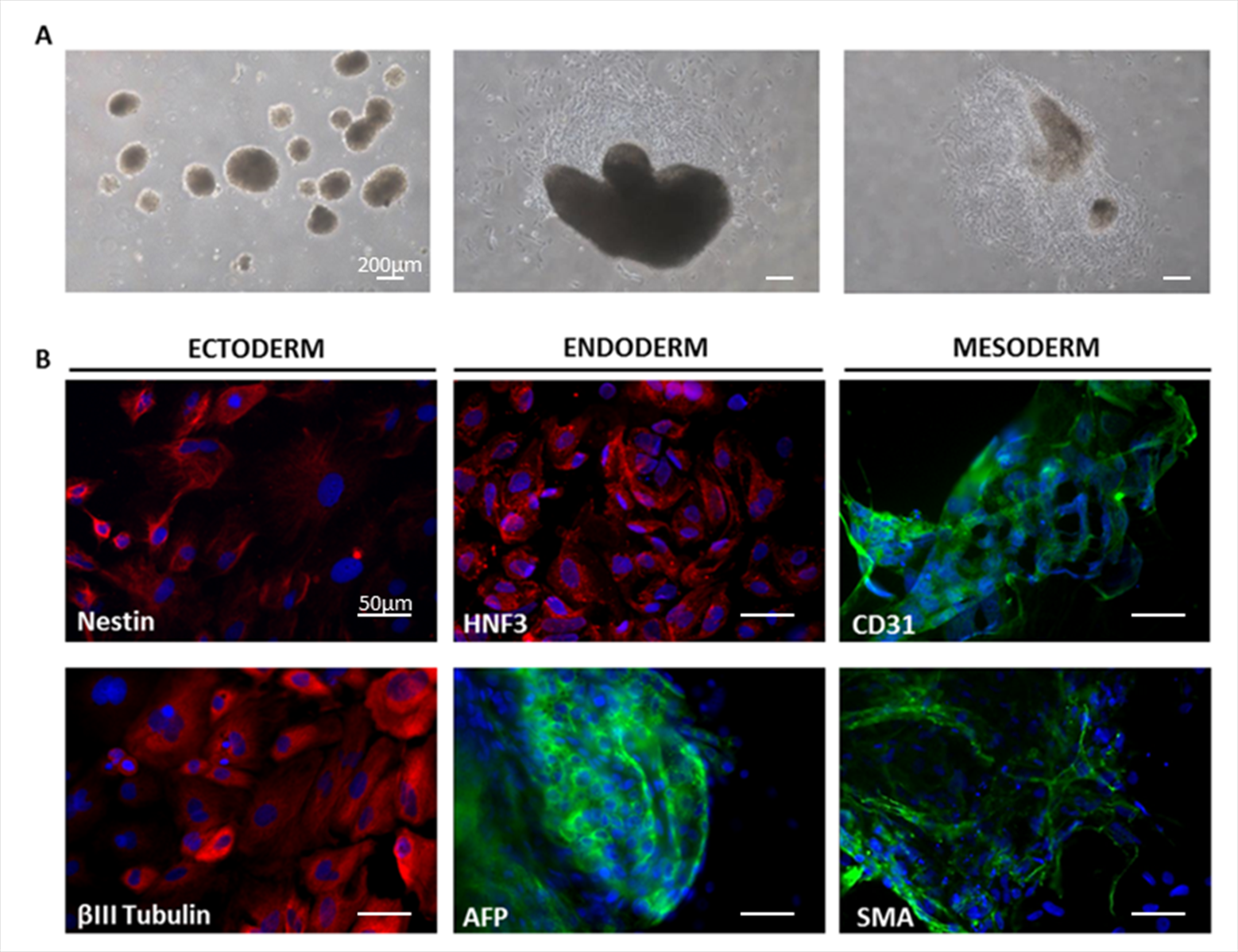

Supplement: S4 Fig — (A) EBs formation after 7 days in ultra-low attachment dish and after 7 days on gelatin with the different morphologies of cells. Scale bars represent 100μm. (B) Immunostaining of iPSC-derived embryoid bodies: Expression of ectodermal (βIII tubulin, nestin), endodermal (AFP, HNF-3β) and mesodermal (CD31, SMA) derivatives. Scale bars represent 50μm. (TIF) [file pone.0152993.s004.tif]

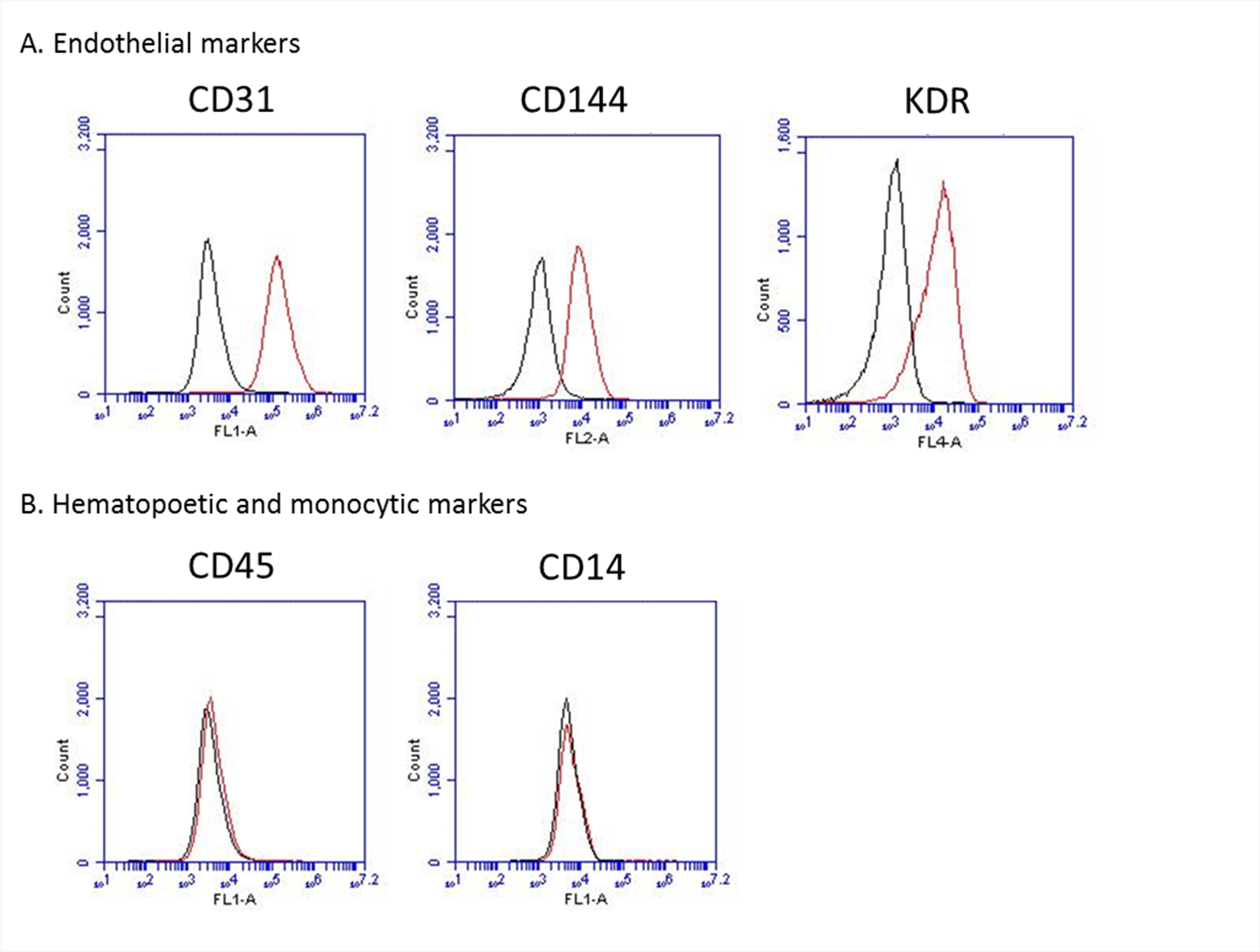

Supplement: S5 Fig — Representative Flow cytometry analysis of the positive endothelial markers CD31, CD144 and KDR (A) and of the negative hematopoetic/monocytic markers CD45 and CD14 (B) (IgG isotopic control: black line, markers: red line). (TIF) [file pone.0152993.s005.tif]
