# Supplementary material for: A Novel Molecular and Functional Stemness Signature Assessing Human Cord Blood-Derived Endothelial Progenitor Cell Immaturity
Source: PLoS One. 2016 Apr 4;11(4):e0152993. doi: 10.1371/journal.pone.0152993 (PMC4820260; doi:10.1371/journal.pone.0152993)
Supplement: S1 Table — (DOCX) [file pone.0152993.s006.docx]

**Table S1. Accession numbers of TaqMan^®^ (Applied Biosystems) assays used for quantitative-RT PCR.**

| Genes | **Taqman Assays IDs** |
| --- | --- |
| CD31 (PECAM-1) | Hs00169777_m1 |
| CD34 | Hs00990732_m1 |
| CDH5 (VE-Cadherin) | Hs00901463_m1 |
| DNMT3B | Hs00171876_m1 |
| GBX2 | Hs00230965-m1 |
| GDF3 | Hs00220998_m1 |
| GRB7 | Hs00917999_g1 |
| ISL1 | Hs00158126_m1 |
| KDR (FLK1/VEGFR-2) | Hs00911700_m1 |
| NANOG | Hs02387400_g1 |
| PODXL | Hs01574644_m1 |
| SOX2 | Hs01053049_s1 |
| TDGF1 | Hs02339499_g1 |
