# Supplementary material for: A Novel Molecular and Functional Stemness Signature Assessing Human Cord Blood-Derived Endothelial Progenitor Cell Immaturity
Source: PLoS One. 2016 Apr 4;11(4):e0152993. doi: 10.1371/journal.pone.0152993 (PMC4820260; doi:10.1371/journal.pone.0152993)
Supplement: S2 Table — (DOCX) [file pone.0152993.s007.docx]

**Table S2**. **Primer sequences of endogenous, exogenous stem cell and endothelial genes used for SYBR assays.**

| Gene | Sequence FWD | Sequence REV |
| --- | --- | --- |
| CD144 | CCC ACC GGC GCC AAA AGA GA | CTG GTT TTC CTT CAG CTG GA |
| KDR | AAT ACC AGT GGA TGT GAT GC | CTG GCA TGG TCT TCT GTG AAG |
| TRA-1-60 | TGA CGG AGA CAG CAA ATA GG | CTT GGA CAG AGC GAT GGA G |
| NANOG | ACT GGC TGA ATC CTT CCT CTC | CTC GCT GAT TAG GCT CCA AC |
| SOX2 | ACT GGC GAA CCA TCT CTG TG | AAT TAC CAA CGG TGT CAA CCT G |
| OCT4 | AGC GAA CCA GTA TCG AGA ACC | AAC CAC ACT CGG ACC ACA TC |
| KLF4 | TCG GAT TCT CTG CTC TCC TC | CCT CAT CTT CTT GTT CCT CCT C |
| C-MYC | CCT AAA TGA TGG TGC TTG GTG | AGG TCA TAA ATG TTG ATC GGA AG |
| EXOGENOUS OCT4 | CCT CAC TTC ACT GCA CTG TA | TCC TGT CTT TAA CAA ATT GGA CT |
| EXOGENOUS KLF4 | GAT GAA CTG ACC AGG CAC TA | TCC TGT CTT TAA CAA ATT GGA CT |
| EXOGENOUS SOX2 | CCC AGC AGA CTT CAC ATG T | TCC TGT CTT TAA CAA ATT GGA CT |
| EXOGENOUS C-MYC | AAG AGG ACT TGT TGC GGA AA | TCC TGT CTT TAA CAA ATT GGA CT |
| GAPDH | AGC AAG AGC ACA AGA GGA AGA G | TCT ACA TGG CAA CTG TGA GGA G |
